# Long-term follow-up of the VORTEC technique: A clampless, sutureless technique for supra-aortic debrancing

**DOI:** 10.1016/j.jvscit.2026.102225

**Published:** 2026-03-12

**Authors:** Michael Hofmann, Mario Lachat, Adrian Palma, Lyubov Chaykovska

**Affiliations:** a Department of Cardiac Surgery, University Hospital Zurich, Zurich, Switzerland; b Hirslanden Clinic Zurich, Zurich, Switzerland; c Docstation, Zurich, Switzerland; d Hirslanden Klinik im Park, Zurich, Switzerland

**Keywords:** Aortic arch, Hybrid repair, TEVAR, Debranching

## Abstract

Hybrid procedures combining open revascularization techniques for supra-aortic branches and aortic stent grafting represent alternatives for aortic arch pathologies. The Sutureless Telescoping Anastomotic Technique-Viabahn Open Revascularization TEChnique (STAT-VORTEC) simplifies debranching. In 55 patients (2008-2018), STAT-VORTEC was performed on 127 of a 148 vessels for debranching. The technical success rate was 100% with no graft occlusion within the first 30 days. Over a mean follow-up of 45 months, the occlusion rate of the STAT-VORTEC was <1%. Perioperative mortality was 7.3%. Neurological complications were noted at 9% with only one complication after STAT-VORTEC. The STAT-VORTEC is an easy-to-learn technique with good long-term patency.

The standard approach for complex aortic arch pathologies has long been open reconstruction using cardiopulmonary bypass and hypothermic circulatory arrest. Decision-making should weigh the perioperative risks, because aortic arch surgery is associated owing to the high invasiveness with higher rates of mortality and stroke than in surgery of the ascending and descending aorta.^1^, ^2^, ^3^, ^4^, ^5^

According to the Penn classification, there are three types of approaches to aortic pathologies, which differ considerably in their surgical invasiveness and range from (I) supra-aortic debranching (SADB) only, eventually in combination with (II) replacement of the ascending aorta and to (III) frozen elephant trunk.^6^ The so-called hybrid procedures combine open revascularization techniques for supra-aortic branches and aortic stent grafting^7^^,^^8^

The Sutureless Telescoping Anastomotic Technique-Viabahn Open Revascularization TEChnique (STAT-VORTEC) is intended to further simplify the SADB and increase anastomosis quality. Our first experiences were published in 2010 and demonstrated excellent short-term results.^9^

Despite various modifications in the meantime,^10^^,^^11^ there has been little research into the long-term results of this technique.^12^ The aim of this study was, therefore, to evaluate the long-term results of SADB using the STAT-VORTEC method in the hybrid treatment of pathologies of the aortic arch.

## Methods

This study was performed retrospectively in accordance with the institutional ethics committee (KEK BASEC No. 2019-00,432). All patients provided informed written consent for the procedures and the analysis of all procedural data.

Major inclusion criteria were multidetector computed tomography (CT)-determined various aortic pathologies, including the aortic arch (Table I), and indication for treatment, including SADB. Outcomes and events were reported according to the end points.

**Table I:** Main aortic pathologies and indication for treatment in our population (n = 55): Some patients show overlapping diagnoses

Characteristics	No.
Aortic arch aneurysm	
Aneurysm extended to ascending aorta (zones 0-3)	36
Aneurysm extended to mid descending aorta (zones 1-4)	31
Subclavian artery aneurysm	3
RSA	1
Abberant RSA	2
Dissection	10
Type A	0
Type B	10
Other arch pathologies	
PAU, floating thrombus, Kommerell's diverticulum	6

*PAU*, Penetrating aortic ulcer; *RSA*, right subclavian artery.

All data are available and obtained from the clinical information system of the University Hospital of Zurich (Dendrite Clinical System and KISIM 5.0.9.3). The data were compiled in Excel (Microsoft Corporation) and prepared for statistical evaluation.

The review core laboratory for the CT data included an independent radiologist as well as a senior surgeon.

### Patient population

The 55 patients (18 female, 37 male) presented underwent surgery for their aortic pathologies from January 2008 to December 2018. In all included patients, STAT-VORTEC was performed for SADB, but not exclusively. Especially in larger vessels (eg, the brachiocephalic trunk) with straightforward exposure, an open anastomosis technique was also used.

The exclusion criteria for STAT-VORTEC are outlined in Table II. Patient baseline characteristics are summarized in Table III. In four cases, it was a redo operation. All operations were performed without cardiopulmonary bypass.

**Table II:** Exclusion criteria for Sutureless Telescoping Anastomotic Technique-Viabahn Open Revascularization TEChnique (STAT-VORTEC)

Contraindications	Alternatives
Absolute	
Massive calcification of the supra-aortic vessels/aorta (risk of embolism !)	Extrathoracic SADB in noncalcified segments
Connective tissue disorders	Open surgery with pledgeted Teflon-reinforced sutures
Relative	
Difficult exposure of supra-aortic branches - eg left subclavian artery (excessive manipulations of aortic arch pathologies should be avoided)	Endovascular treatment (eg, branched stent grafts or periscope technique) or extrathoracic SADB

*SASB*, Supra-aortic debranching.

**Table III:** Baseline characteristics for all patients

Characteristics	Values	*P* value
No of patients	55	
Age, years	72 ± 8 (range, 56-87)	
Sex		
Male	37	0.68
Female	18	0.32
History of		
COPD	31	0.56
Smoking	24	0.44
Arterial hypertension	41	0.75
Diabetes mellitus	6	0.11
PAD	7	0.13
ASA class		
III	47 (86)	
IV	7 (13)	
V	1 (1)	
Euro Score	1.86-33.06 (median, 5.2)	
Follow-up, years	45.12 ± 23.0 (range, 0-86)	
>2	45 (82)	
>3	36 (65)	
>4	23 (42)	

*ASA*, American Society of Anesthesiologists; *COPD*, chronic obstructive pulmonary disease; *PAD*, peripheral artery disease.

Values are mean ± standard deviation or number (%) unless otherwise indicated.

### The surgical concept

The approach to patients with aortic arch pathologies was in most cases a multistage treatment procedure. The first step is the creation of an appropriate landing zone (zone 0-Ishimaru) for endovascular therapy. In our cases, this step is achieved by median sternotomy and off-pump SADB (STAT-VORTEC; Penn classification I). The second step is the implantation of an endovascular stent prosthesis anchored in zone 0.

The diameter of the ascending aorta varied greatly among the different pathologies (range, 28-65 mm; median, 4.4 mm). To prepare the ascending aorta for the insertion of an endovascular prosthesis with a maximum diameter of 45 mm and necessary oversizing of 15% to 20%, an epiaortic wrapping procedure was performed in 46 cases.^13^^,^^14^ This procedure reduced the diameter to the desired 35 mm.

**Fig 1:**
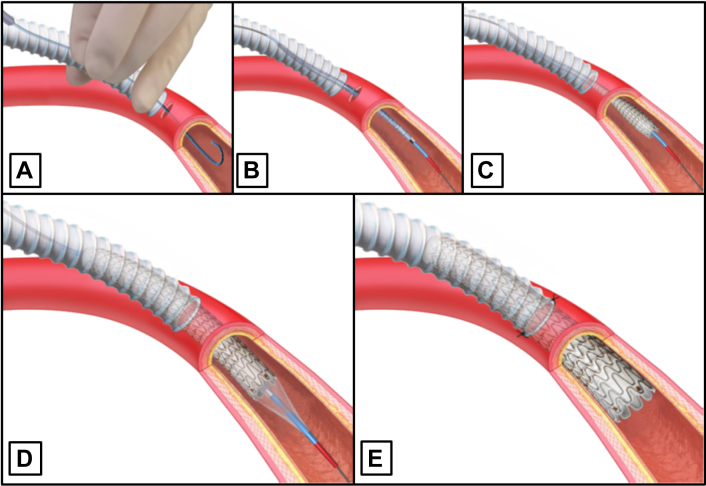
Sutureless telescoping anastomotic technique: Viabahn open revascularization TEChnique. **(A)** Puncture of the Vessels and Insertion of the guide wire (Rose wire) through the graft. **(B)** Introduction and **(C)** deployment of the Viabahn. **(D)** Dilatation using a percutaneous transluminal angioplasty balloon. **(E)** Final result.

The surgical STAT technique (Fig 1) has been described in detail previously and can be used for difficult anatomical vascular accesses or after previous operations with significant adhesions or scare tissue.^9^^,^^15^^,^^16^ In our patients (SADB), the technique is used for distal anastomosis of a vascular graft (ring-enforced polytetrafluoroethylene graft, JOTEC) and the native supra-aortic branch. We always start with the proximal (aortic) anastomosis. Once the distal anastomosis has been completed, blood flow can be restored immediately, reducing the ischemia time to a minimum.

**Fig 2:**
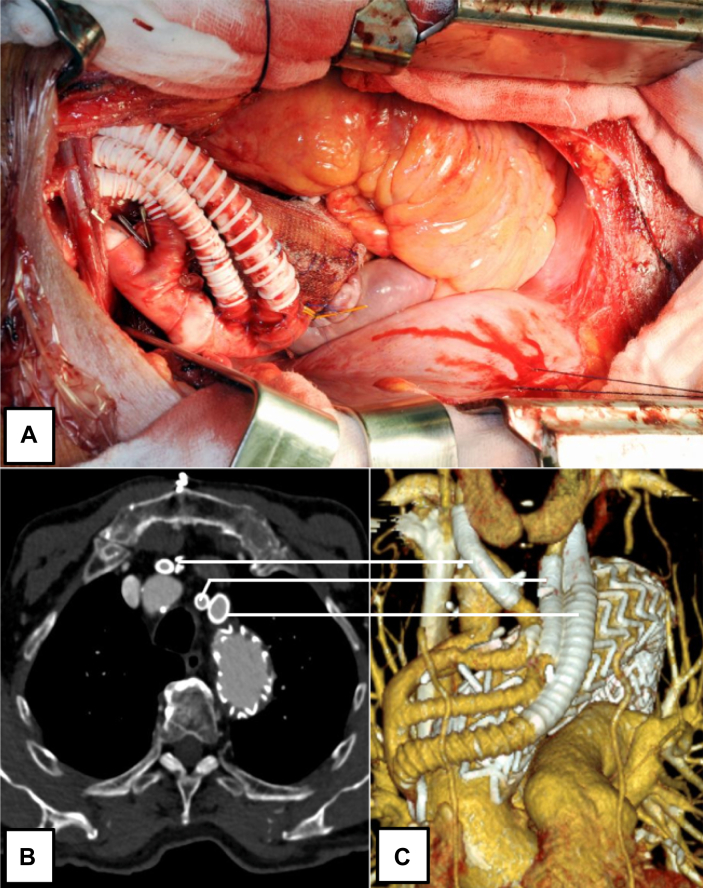
Postoperative computed tomography (CT) scan. **(A)** Intraoperative view. **(B** and **C)** Three-dimensional reconstruction) postoperative CT scan 129 months later.

The proximal anastomosis (lateral-terminal) was performed on the right side of the aorta at the level of the right atrium (proximal zone 0b) (Fig 2, *A* and *C*). Proximal anastomosis with the ascending aorta is sutured in an open surgical fashion. For this purpose, the aorta was clamped tangentially after systemic heparinization (100 IU/kg body weight).

STAT-VORTEC anastomosis can be performed as end-to-side or as end-to-end technique. Despite their simplicity, exact knowledge of the anatomical situation (eg, branches) is indispensable. Fig 1 describes the principle of the technique in end-to-side anastomosis.

As the first step of STAT-VORTEC, a healthy vascular segment is punctured. The dissection of the vessel in the entire circumference or for the placement of a vascular clamp is not necessary.

After the puncture, a soft wire (Rosen wire, Cook Medical) is inserted into the vessel according to the Seldinger technique (Fig 1, *A*). While the wire is fixed at the tip, the end of the wire is passed out through the graft in a retrograde fashion. The first surgeon is responsible for the step-by-step sequence of the STAT. A second surgeon pays meticulous attention to ensure that there are no dislocations or accidental injuries caused by unintentional wire advancement.

During SADB, a wire is guided through a graftotomy. Once the distal anastomosis is complete, blood flow is restored. Then, the lateral opening is closed with the U-shaped suture that was applied beforehand.

**Fig 3:**
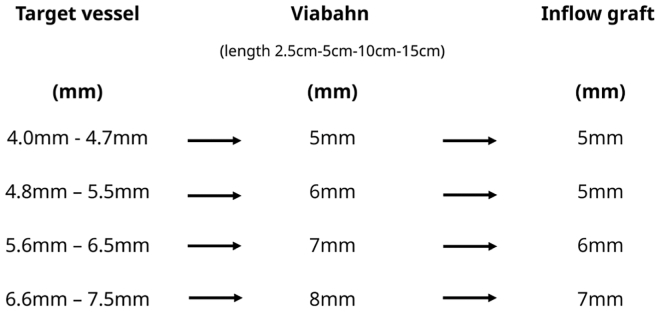
Selection of the appropriate Viabahn and Inflow graft after sizing of the target vessels (computed tomography [CT] scan).

In the next step, a Viabahn (W. L. Gore & Associates) is inserted (Seldinger technique) guided by the wire (Fig 1, *B* and *C*). Currently, we use Viabahn at a length of 5 cm. The decision for the appropriate size/diameter is made after previous measurement (CT scan) and should slightly oversized (1-2 mm) (Fig 3).

Approximately 50% of the Viabahn is placed in the vessel, and 50% is placed in the supplying vascular graft prosthesis. The puncture site should be chosen in such a manner that an occlusion of smaller branches (eg, subclavian artery or vertebral artery) is avoided.

Subsequently, and after the vascular prosthesis has been placed at the anastomotic site, the Viabahn is released and retrogradely de-aired.

Postdilatation is performed using a percutaneous transluminal angioplasty balloon of the same size. This step is particularly important in the setting of scar tissue and guarantees complete unfolding of the Viabahn (Fig 1, *D*). Additional stitches for fixation (2-3 single stitches) complete this procedure (Fig 1, *E*).

The operating times were 160 to 585 minutes (median, 267 minutes).

### Postoperative anticoagulation

Postoperative anticoagulation consisted of intravenous therapeutic heparinization (0.4-1.0 anti-Xa IU/mL) with aspirin (100 mg/d). In the early years of the period evaluated, long-term therapy consisted of a vitamin K antagonist (international normalized ratio of 2.0-2.5) in combination with aspirin (100 mg/d). In line with updated guidelines, since 2011 dual antiplatelet therapy with aspirin (100 mg/d) and clopidogrel (75 mg/d) was later implemented (or in cases of contraindications for vitamin K antagonists).^17^ The combination of rivaroxaban (20 mg/d) and aspirin (100 mg/d) was used in patients from 2018 onward.^18^

### Follow-up and end points

After SADB, the follow-up CT scan protocol includes arterial and venous phases. The first examination should take place immediately after surgery for a postoperative check, followed by another examination within the first year (ideally after approximately 6 months, in accordance to further treatment approaches). After that, annual checkups are scheduled (Fig 2). The CT images were reviewed by the senior radiologist in charge as well as by a senior vascular surgeon.

**Fig 4:**
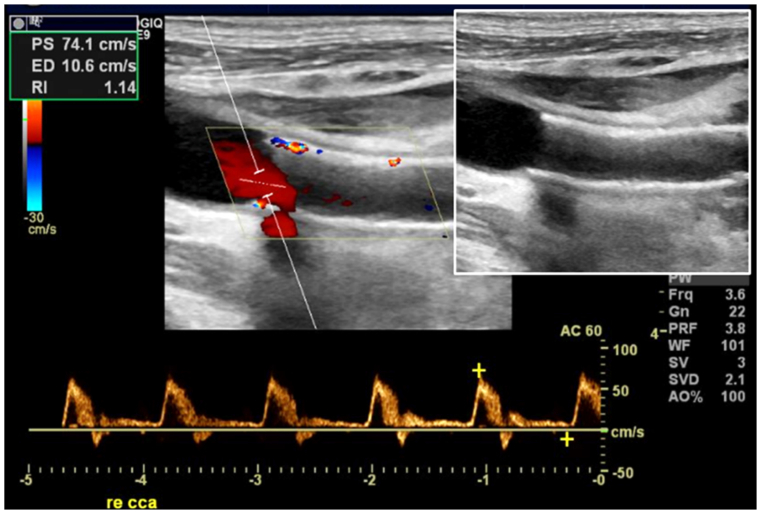
Postoperative duplex ultrasound image (small window: without color).

Postoperative results were also documented by postoperative duplex ultrasound examination of the supra-aortic branches in case of clinical suspicion and/or clinical signs of malperfusion (Fig 4).

The primary end points were all-cause mortality, as well as perioperative and long-term neurological outcomes and STAT-VORTEC patency rates.

### Statistical analyses

The analysis was performed retrospectively on all data available for these patients. The preoperative, intraoperative, and postoperative clinical and radiological data were analysed (Dendrite Clinical System and KISIM 5.0.9.3). Descriptive data are presented as mean ± standard deviation (range), if appropriate. Nominal data are provided as counts and percentages. Kaplan-Meier methods (IBM SPSS Statistics 26) were used to estimate overall survival and primary patency rates.

## Results

STAT-VORTEC was used for SADB in 55 patients. Analyzing the supra-aortic vessels separately, a total of 148 vessels (on average, 2.7 vessels/patient) were treated. In total, 127 vessels were treated with the STAT-VORTEC method, and 21 vessels were treated by conventional anastomosis techniques (Table IV).

**Table IV:** Distribution of the Sutureless Telescoping Anastomotic Technique-Viabahn Open Revascularization TEChnique (*STAT-VORTEC*) and open surgical anastomosis/percentage of neurological complications

	VORTEC	Open surgical	VORTEC/SADB	VORTEC
TBC	20	14	58.8	36.4
RCCA	40	1	97.6	72.7
LCCA	51	1	98.1	92.7
LSA	16	5	76.2	29.1
Total (n = 55)	127	21		

*LCCA*, Left common carotid artery; *LSA*, left subclavian artery; *RCCA*, right common carotid artery; *SADB*, supra-aortic debranching; *TBC*, brachiocephalic trunk; *VORTEC*, Viabahn Open Revascularization TEChnique.

SADB with STAT-VORTEC was most frequent used for carotids (right common carotid artery [CCA], 97.6%; left CCA, 98.1%). The left subclavian artery was rarely treated by this method (29.1%). Thus, in 85.8% (n = 127/148) of cases, the SADB was performed with STAT-VORTEC. The procedure was performed safely in all patients (100% technical success) with no graft occlusion within the first 30 days.

In all patients, postoperative CT scan with angiography was performed after SADB and thoracic endovascular aortic repair, to document the outcomes, according to the follow-up CT scan protocol. All Viabahns were fully deployed and sharply demarcated. No kinking, stenosis, or dissection of the stent graft was reported.

During a mean follow-up of 45.12 ± 23.0 months (range, 0-86 months), two patients developed a graft complication (Table V). The reasons for this were an occlusion of the STAT-VORTEC connection in an LCCA and a high-grade stenosis of the proximal aortoprosthetic (open surgical) anastomosis. In this latter patient with Takayasu disease, the bypass graft was successfully recanalized by angioplasty and stent implantation. Consequently, the occlusion rate of the STAT-VORTEC was <1% (1/127).

**Table V:** Postoperative complication (Sutureless Telescoping Anastomotic Technique-Viabahn Open Revascularization TEChnique [*STAT-VORTEC*] and open surgical anastomosis)

Complications	Value
Immediate technical success	100%
Early graft occlusion	0
Late graft occlusion	1
STAT-VORTEC anastomosis	1
Open surgical anastomosis	0
Perioperative mortality	4 (7.3)
Postoperative bleeding	1
Retrograde dissection	1
Multiorgan failure	1
Stroke/cerebral bleeding	1
Early complications (<30 days)	6 (10.8)
Cardiac tamponade	4
Pneumothorax	2
Late complications (>30 days)	2 (3.6)
Graft infection	1
Descendens dissection	1
Neurological complications	5 (9.0)
Permanent	3
Transient	2

Values are number (%).

**Fig 5:**
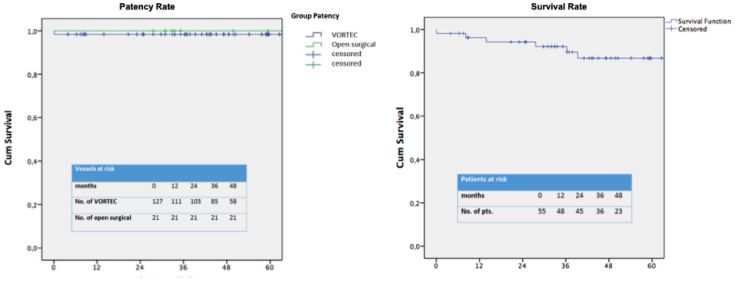
Survival and patency rates.

Perioperative mortality (SADB + thoracic endovascular aortic repair, <30 days) was 7.3% (4 patients). Causes were bleeding complications in the context of multiorgan failure, retrograde dissection, multiorgan failure caused by pneumonia, and a severe stroke with subsequent cerebral hemorrhage (STAT-VORTEC group). The long-term survival rate is shown in Fig 5.

Neurological complications were noted in five patients (9%). Two patients exhibited only temporary impairments followed by full recovery (patient 1, 20 days later amaurosis fugax—complete recovery; patient 2, transient ischemic attack on postoperative day 1—complete recovery within 24 hours). Three patients had a permanent neurological deficit (patient 1, thromboembolic stroke of the left hemisphere after STAT-VORTEC RCCA and LCCA; patient 2, disabling stroke [conventionally sutured anastomosis territory] that was fatal; patient 3, hemisyndrome [conventionally sutured anastomosis territory] on postoperative day 2 with paraparesis of the left upper extremity).

Regarding the technique used for the anastomoses, there was one permanent neurological complication after the STAT-VORTEC and two patients with conventionally sutured anastomosis. All complications in our population were summarized in Table V.

## Discussion

The aim of this study was to present our long-term experience of STAT-VORTEC in patients after SADB.

### Value of STAT-VORTEC

We regard the sutureless telescoping anastomotic technique as a safe procedure that helps to simplify an anastomosis by reducing technical difficulties and invasiveness,^16^ an experience described also by other colleagues.^12^^,^^19^^,^^20^ However, the technique is fully integrated in the surgical repertoire. The technique has been shown to be a significant advancement in the treatment of thoracoabdominal and pararenal aortic aneurysms.^21^ Because of the simplification, this technique also represents an alternative in critical scenarios.^22^^,^^23^

The need to develop alternative procedures results from the considerable complication rates of conventional surgical procedures in aortic arch pathologies, not rarely cases anatomically unfit for standard endovascular techniques such as fenestrated/branched endovascular aortic repair, and therefore more challenging than in the past. Despite numerous improvements, open surgery is associated with a high mortality rate (5%-20%) and neurological complication rates (5%-18%).^1^^,^^3^^,^^24^, ^25^, ^26^

The so-called hybrid procedures combine open revascularization techniques for supra-aortic branches and aortic stent grafting.^7^^,^^8^ The procedures follow the principle of simplification and adaptation of the technique to the technical challenge. The aim is to keep the summation of risks as low as possible by using techniques that are less invasive. Our approach involves the reduction of a complete open surgical procedure in the creation of an adequate landing zone for endovascular therapy.

Open surgical SADB can be performed via a median sternotomy. The debranching procedure described here also does not require an aortic clamp or cardiopulmonary bypass.^9^

The significance of STAT-VORTEC lies in the simplification of distal vascular anastomosis. A difficult exposure with numerous manipulations (dissection and clamping of the vessel) can be avoided and can be change to a no-touch technique.^16^ This idea is more important in the presence of pathological changes in the outlet area (plaque, thrombus) of the supra-aortic branches. Other authors describe in this context the manipulation of supra-aortic vessels in combination with pathological findings as the source of embolization.^27^ However, SADB reduces the risk of thromboembolic events during the endovascular treatment of the aortic arch/descending aorta.

### Advantages of the STAT-VORTEC

#### Primary patency

There are few studies on anastomosis quality and patency rates that also analyze long-term results.^28^ The published results for conventional anastomoses show patency rates of 96% to 100% after a mean of 14.0 to 21.4 months of follow-up.^28^, ^29^, ^30^ In extra-thoracic SADB procedures, comparable good primary patency rates of 95% at 36 months and 92% at 5 years can be demonstrated.^31^^,^^32^

The technical success rate in our population was 100% intraoperatively. The 30-day patency rate of STAT-VORTEC was 100% and follow-up patency rate at a mean of 45 months was 97.8% (1 of 46 patients; 84% follow-up). Our long-term follow-up results confirm that this anastomosis is a reliable alternative to the sutured anastomosis with excellent long-term patency.

#### Reduction of ischemia time

The technique can also be performed quickly with minimal exposure; the described procedure time is 3 minutes (range, 1-9 minutes),^9^ similar to other groups^19^ or experimental studies.^12^

Owing to the anatomical end-to-side but functional end-to-end anastomosis of the VORTEC-STAT, the native antegrade blood flow is only interrupted by the deployment of the Viabahn. Thus, for the vessels, especially the head vessels, the ischemia time only includes the release of the stent graft and postdilatation^16^ and raises the chance to achieve a significant avoidance of organ dysfunction.^20^

#### Hemostasis

A further advantage we see is in the immediate hemostasis, which is achieved by a slight oversizing and final balloon dilatation. These experiences are also confirmed by other authors who describe this technique as an alternative in combination with other procedures.^19^

### Outcomes with STAT-VORTEC

The 30-day mortality rate of our population, at 7.3%, shows a very good result compared with other authors who describe rates of 6% to 10%.^27^^,^^32^ The survival rate of our patients is 84% in the follow-up of 45 months (range, 1.4-133.2 months).

### Comparison with early and long-term results: STAT-VORTEC

There is increasing standardization within our team, which stabilized the outcome of our patients. After the introduction of this technique in our team, total mortality was subsequently reduced from 15% in 2010 (3/20 patients) to 7.3% in 2015 (4/55 patients).^9^ Given the complexity of the procedures, this cannot be attributed exclusively to the SADB. Parallel to the decrease in overall mortality, an improvement in the neurological complication rate of 15% to >9% can also be observed.^9^

In this context, our results are comparable with the systematic review by Moulakakis et al,^33^ who showed a 30-day/in-hospital mortality rate of 11.9%, cerebrovascular event rate of 7.6%, and irreversible spinal cord injury rate of 3.6% with hybrid aortic arch replacement.

Ultimately, it must be said that comparability is difficult owing to the various techniques (with and without cardiopulmonary support), different landing zones (Ishimaru zones 0, 1, and 2), and different strategies used (single stage vs staged procedures).

### Comparison: STAT-VORTEC for reno-visceral arteries

The use of this technique in renovisceral debranching may allow further conclusions to be drawn about the patency of these anastomoses. The primary cumulative patency rate of the STAT-VORTEC in patients with renovisceral debranching after a median follow-up of 22.1 ± 12.9 months was 97%.^34^ These results are similar to and comparable with the outcomes of sutured anastomoses in patients with renovisceral debranching.^35^ A retrospective evaluation of the same population based on CT angiography and magnetic resonance angiography revealed a high remaining cumulative patency rate (84.7 ± 5.2% at 89 months of follow-up) and no stent graft fractures or secondary dislocations.^36^ In 3 of the 34 patients (8.8%), thromboses with stenosis of >50% occurred.

### Future prospects

Certainly, the results for renovisceral arteries cannot be transferred to the SADB procedure. However, they do allow for an overview of what risks exist and must be anticipated. Thus, a better understanding of the formation of thrombi in the transition zone (native vessels-stent graft) seems important. The histological evaluation of self-expanding stent grafts gives rise to the suspicion that continuing dual antiplatelet therapy might be necessary,^37^ and our anticoagulation regime may need to be reconsidered.

### Limitations

One of the limitations of this study is the retrospective character with a small population. Follow-up is not 100%, which is due to the postoperative controls of the patients in their residential areas; this issue has been addressed in another publication.^34^ Therefore, we can make statements about the clinical condition of the patient, but no detailed statements about thrombotic deposits in the Viabahn. The procedures performed are complex. Mortality and neurological outcomes cannot be assigned to one part of the procedure.

## Conclusions

The sutureless telescoping anastomotic technique reduces the technical difficulties and invasiveness of aortic surgery, because clamping or circumferential dissection is not necessary. Moreover, a reduction in the ischemia time is achieved. The analysis of the long-term results in SADB shows an excellent long-term patency rate.

More experience and even longer-term results are necessary to widely establish these methods. Nevertheless, this single-center experience is supportive and may be a good background when starting using the technique reported. Finally, a more detailed knowledge of the behavior of stent grafts in the transition zone, the interaction with the coagulation system and the adaptation of the thrombocyte aggregation inhibition is required.

## Funding

None.

## Disclosures

None.
